# Biomechanics and the Thermotolerance of Development

**DOI:** 10.1371/journal.pone.0095670

**Published:** 2014-04-28

**Authors:** Michelangelo von Dassow, Callie Johnson Miller, Lance A. Davidson

**Affiliations:** 1 Department of Bioengineering, University of Pittsburgh, Pittsburgh, Pennsylvania, United States of America; 2 Duke University Marine Laboratory, Beaufort, North Carolina, United States of America; 3 Department of Developmental Biology, University of Pittsburgh, Pittsburgh, Pennsylvania, United States of America; 4 Department of Computational and Systems Biology, University of Pittsburgh, Pittsburgh, Pennsylvania, United States of America; Dalhousie University, Canada

## Abstract

Successful completion of development requires coordination of patterning events with morphogenetic movements. Environmental variability challenges this coordination. For example, developing organisms encounter varying environmental temperatures that can strongly influence developmental rates. We hypothesized that the mechanics of morphogenesis would have to be finely adjusted to allow for normal morphogenesis across a wide range of developmental rates. We formulated our hypothesis as a simple model incorporating time-dependent application of force to a viscoelastic tissue. This model suggested that the capacity to maintain normal morphogenesis across a range of temperatures would depend on how both tissue viscoelasticity and the forces that drive deformation vary with temperature. To test this model we investigated how the mechanical behavior of embryonic tissue (*Xenopus laevis*) changed with temperature; we used a combination of micropipette aspiration to measure viscoelasticity, electrically induced contractions to measure cellular force generation, and confocal microscopy to measure endogenous contractility. Contrary to expectations, the viscoelasticity of the tissues and peak contractile tension proved invariant with temperature even as rates of force generation and gastrulation movements varied three-fold. Furthermore, the relative rates of different gastrulation movements varied with temperature: the speed of blastopore closure increased more slowly with temperature than the speed of the dorsal-to-ventral progression of involution. The changes in the relative rates of different tissue movements can be explained by the viscoelastic deformation model given observed viscoelastic properties, but only if morphogenetic forces increase slowly rather than all at once.

## Introduction

Developing organisms encounter variable environmental conditions. They may be exposed to environmental toxins, limited nutrients, extreme temperatures, etc. We are particularly interested in one of these environmental factors, temperature, since temperature extremes can result in a diverse set of birth defects [1]–[4]. High fever is one of the largest risk factors leading to birth defects. Fevers as high as 38.9°C during the first month of pregnancy have been linked to defects in the heart and specific forms of spina bifida that parallel defects observed in animal models [5]. Since the timing of exposure correlates to early morphogenetic movements that shape the body plan of the early embryo, we considered the role of temperature in the biomechanics of these early movements in the frog *Xenopus laevis*.

The most surprising aspect of temperature is not that it causes defects, but that many ectothermic animals develop normally across a wide permissive range of temperatures. However, the frequency of developmental defects jumps to 100% above or below this range [6]–[9]. Within the permissive range, developmental rates can vary by more than three-fold with temperature, with little change in the frequency of defects. Most studies indicate little or no change in the relative timing of morphogenetic events as developmental rate varies [10]–[16], although differences in the temperature dependence of cleavage stages and embryonic morphogenesis have been observed [7], [11]. To understand why development fails at high and low temperatures, we need to understand the mechanisms that prevent it from failing, despite dramatic variation in developmental rate, across intermediate temperatures.

Temperature is a key regulator of rates of chemical and biological processes during development. Much of this variation can be understood in the temperature dependence of chemical reactions rates such as the rate of the ATPase activity, the rate of myosin cross bridge cycling [17], [18] or the rate of exchange of GDP for GTP on actin monomers and the rates of actin polymerization. Temperature dependence of diffusion may also regulate cellular processes such as signaling and the assembly of multi-protein complexes. Rates of simple reaction and diffusion processes vary smoothly with temperature but complex events, such as progression through the cell cycle, often do not [19].

We hypothesized that coordinating the biochemical processes of patterning with the mechanical processes of tissue deformation and movement is crucial to maintaining normal morphogenesis across the permissive temperature range [20]. Since morphogenesis requires both forces to deform tissues and the establishment of stiff tissues to limit or resist deformation [21], we propose that the rate of morphogenetic movements should depend on the forces driving the movement, the viscoelastic resistance of the embryonic tissue, and the actomyosin contractility underlying these physical processes. Both cellular force generation and viscoelasticity are strongly dependent on temperature [22]–[31]. For example, cultured human alveolar epithelial cells are much stiffer and more solid-like, while exerting twice the traction forces, at 37°C than at 13°C [29]. Therefore, we expected that temperature dependence of developmental rate would require fine control of the mechanics of the embryonic tissue. In this study we formulate biomechanical models of the temperature dependence of morphogenesis and test both the assumptions and consequences of those models within a temperature range that permits normal development.

Gastrulation is one of the earliest and most significant morphogenetic movements in vertebrate development (Movie S1). In the frog, *Xenopus laevis*, gastrulation integrates the action of multiple cell behaviors, including epiboly, involution, convergent extension, and convergent thickening, to close the blastopore over the endoderm and establish the archetypical body plan consisting of the three primary germ layers [32]. The stages of gastrulation are marked by: 1) constriction of bottle cells to encircle the yolk plug; 2) formation of a groove at the site of bottle cell contraction, initially at the dorsal-anterior end of the yolk plug, and progressing to the ventral-posterior end of the yolk plug; 3) initial involution at the dorsal anterior end of the blastopore lip spreading to the ventral-posterior lip; and 4) closure of the blastopore [33]. Blastopore closure often fails when cell motility, cell adhesion, or the cytoskeleton are perturbed. We investigate the temperature dependence of blastopore closure because these movements are easily visualized and exhibit clear milestones.

Biomechanical contributions to the temperature dependence of developmental rate appear necessary to explain how tissue movements and deformations are coordinated with molecular patterning processes within the permissive temperature range. Many other molecular and cellular processes may influence the temperature dependence and temperature limits of development. These include the temperature dependence of cell-cycle regulation [9] or membrane fluidity [34], excessive apoptosis at high temperatures and/or limits to protection by heat shock proteins and other pathways that protect against cell damage [1], [35]–[37]. Here we focus solely on biomechanics and variation in developmental rates over the permissive temperature range.

## Results

### Changes in viscoelasticity and force generation

**Figure 1:**
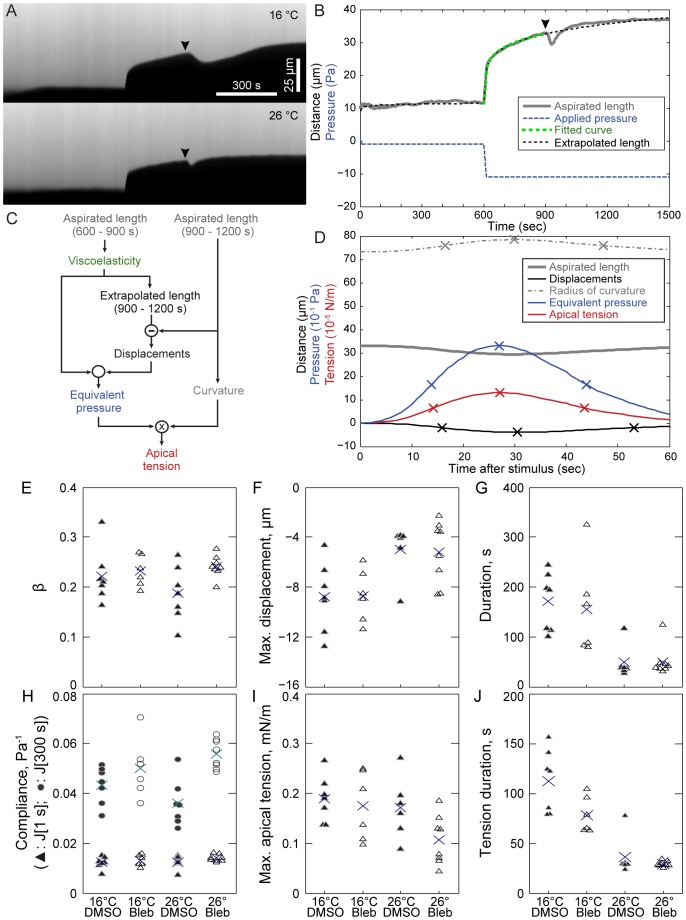
Temperature dependence of compliance and strength of induced contraction. (A) Representative kymographs of microaspiration with electrically induced contractions at 900 seconds at 16°C (upper) and 26°C (lower). (B) Fit of power law viscoelastic model to the aspirated length from 600 to 900 s for the 26°C case. (C) Flow chart for analysis of contractions. (D) Contraction analysis. ‘X's indicate half-max, max, and return to half-max for each curve. Panels B and D show data from the lower embryo in A. Arrowheads in A and B indicate electrical stimuli. (E) β, (F) maximum displacement during induced contraction, (G) duration (half-maximum until return to half-maximum displacement) of contraction, (H) compliance at 1 s (triangles) and at 300 s (circles), (I) maximum apical tension during induced contraction, (J) duration of apical tension. Triangles and circles: individual embryos; X's: means.

To understand the dependence of morphogenesis on temperature we first developed a simple, generalized model (Methods, Model 1; Text S1.1) to predict expected changes in tissue viscoelasticity and force generation with temperature. We then tested the model's assumptions and predictions using micro-aspiration to measure tissue viscoelasticity, and electrically induced contractions to measure force generation (Fig. 1A–D) [38]. Even without considering the complexities of cell adhesion, cell signaling, cytoskeletal dynamics, etc., a three dimensional (3D), non-linear, large-deformation viscoelastic model would involve large numbers of poorly constrained parameters. This would limit its predictive value. A simplified linear, small deformation model allows us to incorporate the essential features (temperature dependence of deformation rates, forces, and viscoelasticity) with a minimum of parameters, all of which can be experimentally constrained.

We formulated our initial model by assuming that the relative timing and magnitude of tissue deformation is identical at different temperatures (Methods, Model 1). This is based on the observations that different developmental stages look similar at different temperatures within the permissive range, and that the proportion of time spent in each stage is similar at different temperatures (e.g. [10]). Thus, we assumed that stages define a temperature-independent “developmental time” that is proportional to clock time through a temperature-dependent constant (see Methods: Model 1, eqn. 5 for definition). For the same reason, we assume that the relative timing of gene expression and protein activation are identical at different temperatures. Furthermore we assume that the timing and magnitude of force generation scales uniformly with temperature: forces exerted by the tissues vary the same way with stage at every temperature, except for possibly a temperature-dependent proportionality factor. This assumption is biologically plausible since we assume force production is controlled by changes in gene expression and protein activity, the timing of which we already assumed varies uniformly with temperature. Furthermore, there are a small number of proteins that contribute to generating force in the embryo (primarily actomyosin complexes [39], [40]) and it is parsimonious to assume that these proteins are affected by temperature in the same way in every cell. Making these assumptions allows us to predict how the viscoelastic properties should change with temperature over the permissive range (Method, Model 1: eqns. 15–16). Specifically, we predicted that the exponent (β; see Table 1 for a list of symbols) in the power law model of viscoelasticity (eqn.14) should be independent of temperature. This parameter indicates whether the tissue behaves more like a solid (β = 0) or a fluid (β = 1).

**Table 1:** Symbols.

c	Proportionality between stress at T_2_ and stress at T_1_ in generalized model
f	Peak apical tension during contraction
h	Duration of apical tension during contraction
J	Compliance: relates strain and stress as a function of time since stress application
k	Hypothetical constant of proportionality between peak apical tension and stress driving blastopore closure
R_CP_	Relative duration of blastopore closure to progression of involution: t_C_/t_P_
t	Clock time
T	Temperature
t_C_	Time for blastopore closure
t_P_	Time for dorsal-ventral progression of involution
w	Slope of stress in ramp model
α	Proportionality between developmental time and clock time in generalized model
β	Exponent that determines time dependence of compliance
ε	Strain (Ln[L/L_0_), a non-dimensional measure of deformation
σ	Stress (force/area)
τ	Developmental time in generalized model (Model 1)

We tested the prediction that β is independent of temperature using microaspiration, and then tested our assumption that deformations during induced contractions (Fig. 1) and normal gastrulation are independent of temperature. We performed microaspiration at 16°C and 26°C and fitted the deformations to a power law model of viscoelasticity (Methods: eqn. 1) to estimate β and J[1] (the compliance – the proportionality between strain and stress – at 1 second after stress is applied). The temperature dependence of cell stiffness and force generation depends on myosin activity in human alveolar epithelial cells [41]. Therefore we tested for effects of inhibiting myosin contractility by applying blebbistatin at these two temperatures.

Consistent with the prediction of our model, there was little or no change in β with temperature (Fig. 1 E; Tables 2 & 3), whereas incubation of the embryo in blebbistatin increased β slightly but statistically significantly, indicating that inhibiting myosin contractility made the tissue slightly more fluid-like (Fig. 1E; Table 2). The interaction between temperature and blebbistatin treatment was not statistically significant for β (Table 2).

**Table 2:** ANOVA table for viscoelasticity and contractions.

	temperature	media	clutch	temperature*media	temperature*clutch	media* clutch
β	P = 0.7	***P = 0.034***	P = 0.4	P = 0.18	P = 0.3	P = 0.8
	F_1,5.1_ = 0.170	F_1,5.3_ = 8.09	F_5,1.8_ = 2.16	F_1,9_ = 2.17	F_5,9_ = 1.53	F_5,9_ = 0.511
Max. Contraction	***P = 0.029***	P = 0.8	P = 0.8	P = 0.9	P = 0.12	P = 0.14
	F_1,5.0_ = 9.107	F_1,5.0_ = 0.0589	F_5,5.9_ = 0.434	F_1,8_ = 0.0378	F_5,8_ = 2.47	F_5,8_ = 2.34
Ln[Duration]	***P = 0.0025***	P = 0.8	P = 0.8	P = 0.6	P = 0.3	P = 0.4
	F_1,5.0_ = 31.0	F_1,5.0_ = 0.0716	F_5,3.2_ = 0.496	F_1,8_ = 0.287	F_5,8_ = 1.50	F_5,8_ = 1.13
J[1]	P = 0.7	P = 0.078	P = 0.3	P = 0.6	P = 0.5	P = 0.6
	F_1,5.1_ = 0.131	F_1,5.2_ = 4.81	F_5,1.4_ = 3.39	F_1,9_ = 0.288	F_5,9_ = 1.02	F_5,9_ = 0.762
J[300]	P = 0.9	***P = 0.020***	P = 0.4	**P = 0.024**	P = 0.3	P = 0.080
	F_1,5.1_ = 0.0075	F_1,5.0_ = 11.3	F_5,5.2_ = 1.2	F_1,9_ = 7.33	F_5,9_ = 1.52	F_5,9_ = 2.87
Max. Apical Tension	P = 0.14	P = 0.3	P = 0.8	P = 0.21	P = 0.3	P = 0.15
	F_1,5.0_ = 3.05	F_1,5.0_ = 1.64	F_5,4.7_ = 0.409	F_1,8_ = 1.83	F_5,8_ = 1.47	F_5,8_ = 2.21
Ln[Tension Duration]	***P = 0.0006***	P = 0.094	P = 0.9	P = 0.4	P = 0.20	P = 0.4
	F_1,5.0_ = 57.4	F_1,5.0_ = 4.25	F_5,3.6_ = 0.297	F_1,8_ = 0.961	F_5,8_ = 1.88	F_5,8_ = 1.08

P values and corresponding F values (with degrees of freedom determined by Matlab). “Temperature” and “media” (blebbistatin *vs.* DMSO control) were treated as fixed factors; “clutch” was a random factor. Statististically significant entries in bold.

**Table 3:** Temperature dependence of mechanical and morphogenetic parameters.

Process	Parameter (X)	X(16°C)/X(26°C) (LB, UB)^1^
Morphogenesis	t_c_ (time for blastopore closure)	**2.73** ** **(2.29, 3.26)**
	t_p_ (time for dorsal to ventral progression of involution)	**3.29** ** **(2.75,3.94)**
	R_CP_ (t_c_/t_p_)	**0.83** ** **(0.72, 0.94)**
Viscoelasticity	Compliance J(1)	0.98 (0.85,1.13)
	Compliance J(300)	1.02 (0.9,1.16)
	β	1.04 (0.88,1.24)
Stimulated contractions	Contraction Duration	**3.36** ** **(2.23,5.06)**
	Contraction Magnitude	**1.75** * **(1.29,2.38)**
	Tension duration (h)	**2.85** ** **(2.26,3.6)**
	Peak Apical Tension (f)	1.37 (0.98,1.94)
Endogenous actin dynamics	Duration^2^	**1.32** ** **(1.10, 1.57)**

*P≤0.05;

**P≤0.01:

Significant difference between 16 and 26°C for log transformed parameters.

1 Estimated mean and confidence interval for log-transformed parameters were determined using ANOVA (mechanics and actin) and Tukey's honestly significant difference criterion, or T-tests (morphogenesis).

Values were then transformed back to a linear scale to provide estimates of the lower and upper bounds (LB, UB) on the ratio.

2 Comparing 17° and 27°C. Ratios for the durations of actin contractions were 0.9 between 16.9°C and 21.3°C, and 1.5 between 21.3°C and 26.7°C.

To obtain confidence bounds here, we used ANOVA with temperature treatment as a categorical variable, and explant as a random factor; clutch was excluded because it was non-significant.

Our model assumes that the large scale deformations of morphogenesis are independent of temperature (Methods: eqn. 6). Therefore, we expected that small scale deformations associated with cell contractions should also be independent of temperature. To test this in a controlled manner, we electrically induced contractions of tissues in the channel of the microaspirator. Contrary to our predictions, the magnitudes of contractions were reduced by 44% at 26°C compared to contractions at 16°C (Fig. 1A, F; Table 2 & 3). Contractions lasted 3.4 times longer at 16°C than at 26°C (Fig. 1A & 1G; Tables 2 & 3). Blebbistatin did not significantly affect contraction magnitude or duration (Fig. 1F–G, Table 2).

In contrast to results from studies with human cells [29], temperature had little effect on the compliance of the tissue (J[1] or J[300]; Fig. 1A, H, Table 2 & 3). Blebbistatin did not significantly affect the compliance at 1 second, but – due to the change in β – it did increase compliance at 300 s (Fig. 1H, Table 2). The interaction between temperature and blebbistatin treatment was statistically significant for J[300], though not for J[1] (Table 2). Therefore, myosin activity may influence the temperature dependence of stiffness in this system.

From the contraction profiles we can calculate the forces driving induced contractions based on the viscoelastic properties of the tissue (Fig. 1B–D; [38]). Previous results suggest that these forces are best described as apical tensions [38]. Note that our method for calculating apical tensions assumes that the compliance does not change during contractions. We have been unable to test this so far because contractions are transient. However, neither the calculated tensions nor the viscoelastic parameters varied significantly with temperature. Therefore, it is parsimonious to assume that temperature does not substantially affect the true tension or compliance during contractions.

The peak apical tension was not significantly affected by temperature (Fig. 1I; Tables 2 & 3), but the duration of tension was greatly reduced at higher temperature (Fig. 1J; Tables 2 & 3). The higher contraction speed, but unchanged viscoelasticity and unchanged peak force, drive the decrease in contraction magnitude at higher temperature. The small reduction in apical tension and tension duration in blebbistatin treated embryos was not statistically significant (Fig. 1I–J; Table 2). The molecular mechanisms responsible for contraction in response to electrical stimulation in these tissues have not been elucidated, and may depend more on F-actin polymerization-dependent changes in membrane tension [42] than myosin-mediated contractility.

### Temperature dependence of morphogenesis

**Figure 2:**
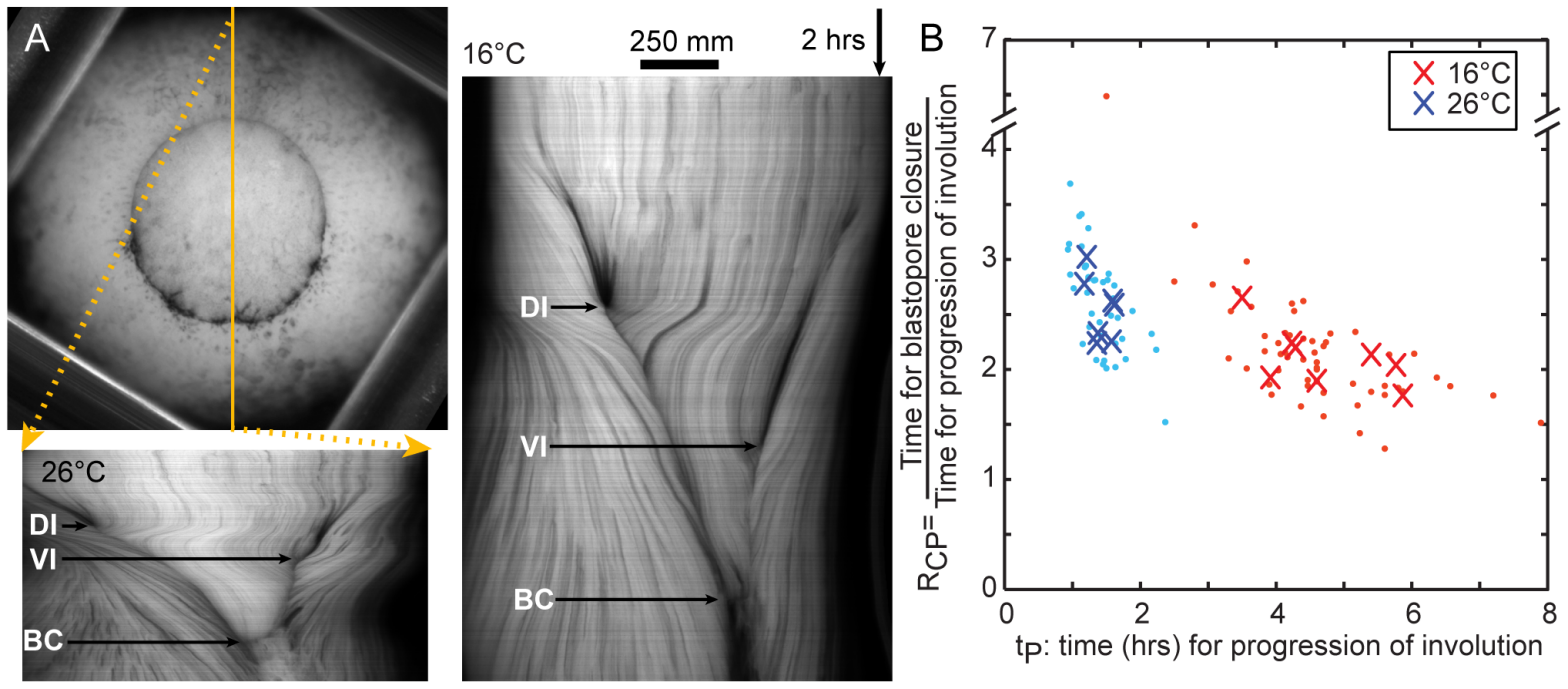
Blastopore closure at high and low temperatures. (A) Upper: vegetal view of an embryo showing the blastopore soon after the start of dorsal superficial involution. Lower left: kymograph of blastopore closure at 26°C, taken along the yellow line from the dorsal side to the ventral side, showing the points when dorsal (DI) and ventral (VI) superficial involution begin, and when the blastopore closes (BC). Right: kymograph taken along a line from the dorsal to the ventral side at 16°C. (B) The ratio (R_CP_) of the time for blastopore closure to the time for dorsal-to-ventral progression of involution versus the time (t_P_) for dorsal-to-ventral progression of involution. Dots indicate individual embryos. X's indicate medians for clutches (4 to 8 embryos each).

Given the failure of our prediction that contraction magnitudes would remain unchanged with temperature, we tested our assumption that the relative timing of morphogenetic events is independent of temperature (Model 1, eqn. 5). To test this we measured how the ratio of the durations of two morphogenetic processes changed with temperature. While chosen primarily due to the clarity of their beginning and end points, these two processes – blastopore closure, and the dorsal-to-ventral progression of superficial involution (Fig. 2A; Movie S1) – also reflect distinct processes. Blastopore closure involves large scale tissue movements, whereas superficial involution is much more local, and likely represents the timing of signaling events that trigger changes in cell behaviors. Therefore the dorsal-to-ventral progression of involution reflects the difference in timing of cell behaviors between the dorsal and ventral side (Fig. 2A).

Contrary to our assumptions, the relative durations of different morphogenetic movements varied with temperature. The time (t_P_) between the beginning of involution on the dorsal side and the beginning of involution on the ventral side was 3.3 times longer at 16°C (n = 8 clutches) than at 26°C (n = 7 clutches). However the time (t_C_) between the beginning of dorsal involution and the completion of blastopore closure was only 2.7 times longer at 16° than 26°C. These changes in the timing of developmental events were similar to the changes in duration of induced contractions (Fig. 1G). The relative duration of blastopore closure (R_CP_), measured as the ratio of t_C_ to t_P_, was significantly reduced at lower temperature, from 2.55±0.29 at 26° to 2.11±0.27 at 16°C (mean ± SD; Fig. 2B; *P = 0.01*; t-test; Table 3).

### The role of the time dependence of force generation

We were curious whether the viscoelastic model of morphogenesis could explain the effect of temperature on the relative durations of developmental processes, specifically on R_CP_. Here we relax the assumption that all developmental processes follow the same clock. We assume instead that “patterning”, including all the molecular processes driving changes in cell behaviors, follows one clock, but that large scale morphogenetic movements deviate from the timing of patterning due to tissue viscoelasticity.

**Figure 3:**
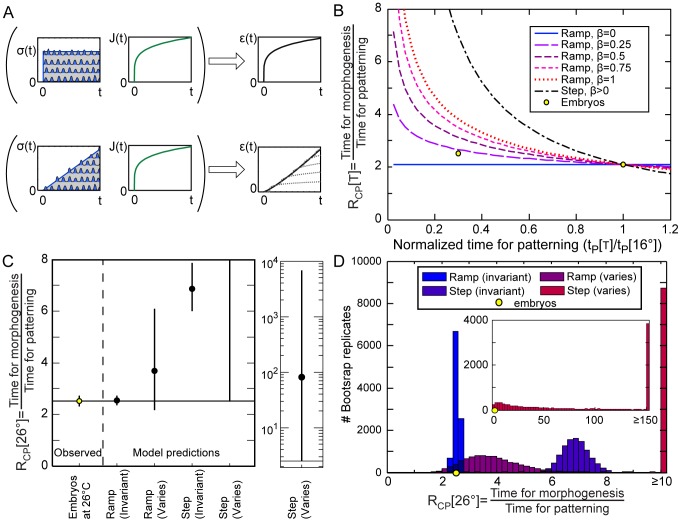
Comparison of viscoelastic models of morphogenesis for ramped versus stepped forces. (A) Diagram of model. Summed contractions (wavy lines) average out to stepped or ramped stresses (σ) depending on when cells begin contracting. When applied to the viscoelastic material with compliance J[t], the deformations (strains, ε) follow the time course of ramped forces more closely than stepped force. This can be visualized as adding up strains due to a series of stepped forces applied over time (dotted lines on right). (B) Predictions for R_CP_, the ratio of the time for morphogenesis (blastopore closure) to the time for patterning (D-V progression of involution), as a function of the time for patterning at temperature T, normalized to the time for patterning at 16°C, for ramped *vs.* stepped models for different values of β. Yellow dots: grand mean of experimentally observed values. The curves automatically converge to the right hand dot (at 16°C) where T_2_ = T_1_ since t_C_ at T_1_ is used to calculate R_CP_ at T_2_. (C) Comparison of the observed R_CP_ at 26°C to the predictions for models with ramped or stepped forces, and with temperature invariant or varying mechanical properties (inset: prediction for stepped force model with temperature dependent mechanical properties on a log scale.) Error bars indicate confidence intervals. (D) Histogram of bootstrap resampling estimates of R_CP_ at 26°C for each model (10,000 resamples total).

To investigate how the dependence of force production on developmental time might alter the temperature dependence of morphogenesis, we compared two specific variants of the linear viscoelastic model (Methods, Models 2A & B). In the step model we assumed the force driving blastopore closure turns on all at once at the beginning of blastopore closure, immediately reaching a peak force and remaining there. In the ramp model we assumed that the forces driving blastopore closure increased gradually with a constant slope, and that the slope was proportional to the rate of patterning. We assume that all cellular responses including gene activation, and cell behaviors, follow the same clock. Blastopore closure is thought to be driven in large part by convergence and extension of the mesoderm, which pulls the ectoderm over the embryo as the mesoderm shortens laterally [43]. The mediolateral cell intercalation behaviors that drive this do not occur simultaneously throughout the dorsal mesoderm, but instead start and spread progressively [44]. Therefore we might expect that the forces driving blastopore closure ramp-upwards with time (Fig. 3A). In both the step and ramp models, we assumed that temperature had the same effect on cell-generated forces driving blastopore closure as it does on the induced contractions.

In these more specific versions of the general model we made the following simplifying assumptions which tie it to our experimental measurements, leaving no free parameters. We assumed that the progression of superficial involution reflects the timing of patterning events rather than mechanical events. Superficial involution is a localized phenomenon and normal dorsal to ventral progression of superficial involution can be reversed by placing embryos in temperature or oxygen gradients [45], [46]. These observations suggest that progression of involution from the dorsal to the ventral side does not involve large scale mechanical interactions around the embryo, but is tied to the local rate of cellular differentiation. Given this assumption, the rate of patterning (e.g. the timing of when cells begin force-generating behaviors) is inversely proportional to the time for the dorsal-to-ventral progression of involution (t_p_). We approximated the complex movements of blastopore closure with a one-dimensional (1D), linear viscoelastic model (Text S1.1). Additionally, we assumed that the viscoelastic parameters measured by microaspiration (5 minute time scale) could describe viscoelasticity at the time scales of morphogenesis (2 to 6 hrs). Finally, we assumed, based on the lack of statistically significant effects from our microaspiration and induced contraction experiment, that β, J[1], and the magnitude of cell generated force are unaffected by temperature.

With these assumptions, the step model with invariant mechanical parameters, predicts that the time for blastopore closure (t_C_) would be independent of the time for progression of involution (t_P_) since neither the force nor the viscoelasticity change. Therefore R_CP_ would increase rapidly as t_P_ goes down (Fig. 3B): at low temperatures the blastopore would close quickly relative to the propagation of cell behaviors driving involution. In addition, R_CP_ would be independent of the how solid or fluid the tissue is (the value of β; Fig. 3B). The predicted value of R_CP_ for the step model at 26°C (based on the ratio at 16°C) is much higher than the experimentally observed value (Fig. 3C).

By contrast, the ramp model predicts a much weaker dependence of R_CP_ on t_P_. Because the driving force increases with time more slowly when t_P_ is large than when t_P_ is small, t_C_ increases with t_P_ (Fig. 3B). In addition, the dependence of R_CP_ on t_P_ varies strongly with the value of β, i.e. with how solid-like or fluid-like the tissue is (Fig. 3B). Using the average value of β for the DMSO control embryos at both temperatures (Fig. 1E), and the value of R_CP_ at 16°C, the predicted value of R_CP_ at 26°C is surprisingly close to the observed value (Fig. 3C).

The absence of statistically significant effects of temperature on mechanical parameters measured by microaspiration and induced contraction does not mean that the observed differences are not real. Including our observed values of each mechanical parameter for each temperature (from the DMSO controls) substantially changes the outcome of the model. When mechanical parameters vary with temperature, the ramp predicts a higher than observed R_CP_ at 26°C (Fig. 3C). However, it is still closer to the observed value than for the step model, and the bootstrap confidence intervals overlap the observed value. When mechanical parameters vary with temperature, the step model predicts an extremely high R_CP_ at 26°C, although the broad confidence interval includes the observed value (Fig. 3C). Furthermore, we note that the step model is extremely sensitive to variation in mechanical parameters, much more so than the ramp model (Fig. 3C–D). Given observed levels of variation in mechanical parameters, the step model predicts larger variation in R_CP_ at 26°C (Fig. 3D) than we observed in live embryos (Fig. 2B).

### Temperature regulates duration of punctuated F-actin contractions

**Figure 4:**
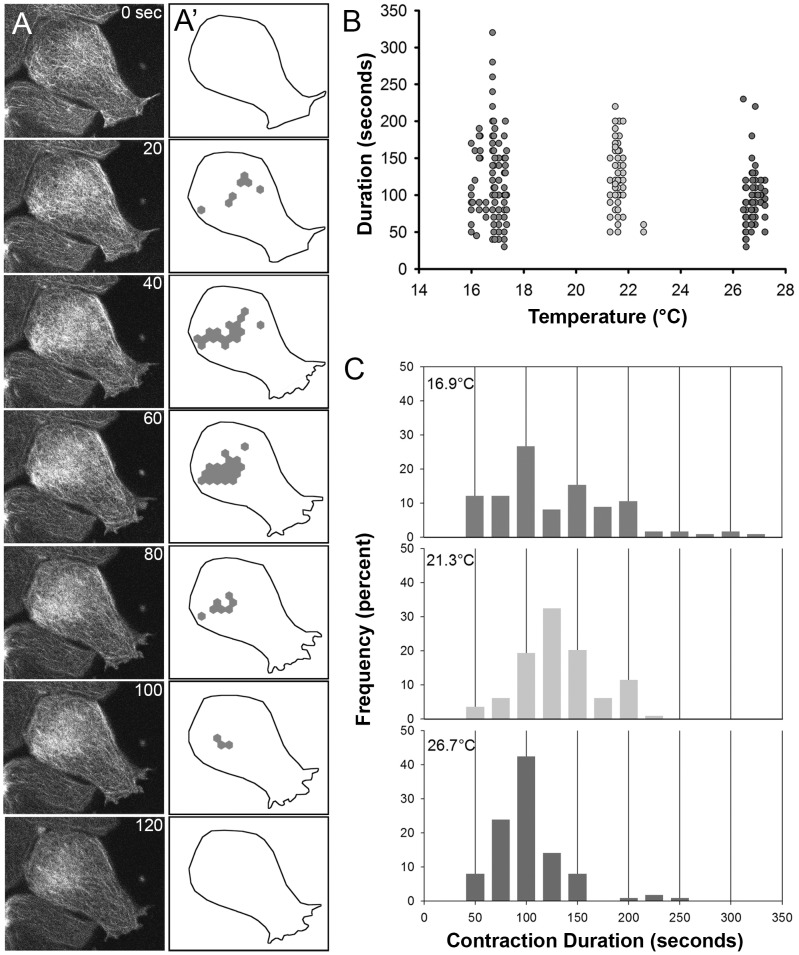
Duration of actomyosin contractions depends on temperature. (A) Sequential frames from a representative time-lapse sequence collected from the basal cortex of an animal cap ectoderm explant cultured on fibronectin-coated glass substrate. F-actin dynamics are revealed in cells expressing the actin-binding domain from moesin coupled to EGFP (moe-GFP) (left column). This sample collected at 16°C. (A′) Schematic of frames matching those in (A) highlighting the cell outline (dotted line) and hexagonal regions of the cell cortex identified as “F-actin contractions.” Regions are categorized as contractions when their integrated intensities are 50% greater than the mean intensity of the basal cell cortex. (B) Duration of individual F-actin contractions across the three temperature regimes. (C) Frequency distribution of the duration F-actin contractions at three temperatures. Note abundant short duration contractions at the low temperature regime.

Recent reports of actomyosin dynamics during morphogenesis (see review [47]) and a previous report by our group that electrically induced contractions are accompanied by a phase of F-actin remodeling [48] suggested that actomyosin dynamics might underlie the complex dependence of morphogenesis on temperature. Furthermore, cortical F-actin dynamics have been implicated in regulating both cell behaviors and biomechanical properties of *Xenopus* embryos at these stages [40], [49]–[51]. Due to technical challenges in recording actomyosin dynamics during microaspiration we investigated actin contractions in the basal cell cortex of ectodermal explants (e.g. animal cap explants). To understand how temperature regulates these dynamics we collected time-lapse sequences of punctuated actin contractions within isolated animal cap explants cultured on fibronectin-coated glass (Fig. 4A). We confirmed the incidence of actin contractions in animal caps at 21.3°C (room temperature) as well as at 16.9°C and 26.7°C (Fig. 4B). Single confocal images did not reveal differences in the qualitative appearance of cortical F-actin across this range of temperatures but time-lapse sequences highlighted consistent retardation of F-actin dynamics at low temperatures and acceleration at high temperatures (see Movie S2).

Quantitative analysis of the duration of F-actin contractions across three temperatures reveal how dynamics of these contractions change as temperatures either increase or decrease from room temperature. As the temperature was reduced from 26.7 to 21.3 to °C the average duration of contractions increased from 92 seconds (+/−34 seconds; n = 113) to 122 seconds (+/−37; n = 114; Fig. 4B). Surprisingly, as the temperature was reduced further, from 21.3 to 16.9°C, the average duration remained essentially unchanged (119+/−58 seconds; n = 124). The non-linear effects of temperature on contraction duration were statistically significant (Table 4). Further analysis of the cumulative distribution for contractions at these three temperatures revealed that the low temperature regime was marked by the addition of many short duration contractions (Fig. 4C). By contrast, the distributions of contraction durations at 21 and 26°C were qualitatively similar, albeit time-shifted by a factor of ∼1.5. We suspected that these short duration contractions might not have been observed at 26.7°C; however, our observations at 21.3°C would have revealed this population had it existed. Confocal observations of actomyosin contractions suggest a redistribution of contraction durations at the lower temperature. Thus, the duration of endogenous F-actin contractions qualitatively paralleled the temperature dependent changes in induced contraction at the high temperature regime but not at the low temperature. The temperature dependence of the duration of individual endogenous F-actin contractions was small relative to temperature dependence of either morphogenesis or the duration of stimulated contractions, but was more aligned with the temperature dependence of the tissue viscoelasticity.

**Table 4:** ANCOVA table for endogenous F-actin contraction duration*.

T^2^	T	clutch	explant(clutch)
**P = 0.0001**	**P = 0.0002**	P = 0.4	**P = 0.02**
**F_1,345_ = 16.6**	**F_1,345_ = 14.2**	F**_10,3.94_** = 1.4	**F_7,345_ = 2.3**

*P values and corresponding F values. Temperature (T) and T^2^ were fixed co-variates; “clutch” and “explant” were random factors, with explant nested within clutch. Statistically significant entries in bold.

## Discussion

Our simple models suggest that a combination of how solid-like or fluid-like the tissue is, and the precise timing of forces driving morphogenesis (all at once, as in the step model, or gradually, as in the ramp model) are critical to determining how well or how poorly morphogenetic processes remain coordinated across a range of temperatures. In contrast, our experimental results suggest that temperature driven variation in developmental rates does not involve either changes in the viscoelastic properties of the embryo, or the magnitude of cell- generated forces, neither of which change with temperature. Instead, the temperature dependence of developmental rate appears to depend on changes in the timing of force generation, and tolerance of variation in the relative rates of different developmental processes (Table 3). The rate of dorsal-to-ventral progression of involution, which we suspect reflects the progression of patterning, increased more quickly with temperature than the rate of blastopore closure, which appears to reflect large scale mechanical interactions [43], [52]. One exceptional example of tolerance to variation in development during gastrulation is described in Text S1.2. This study was conducted within the normal developmental temperature range of *X. laevis*. We hypothesize that developmental defects may result when asynchronies among developmental processes exceed normal tolerance limits.

Because our models are simplified generalizations of the process of morphogenesis they should be applicable to a wide range of morphogenetic processes. However, to make a generalized model, we had to leave out the complications of the real 3D geometry, large strains, material non-linearity, and plasticity. *Xenopus* embryonic tissue stiffens with increasing strain, although tissues exhibit near linear mechanical properties up to fairly large strain [38], [40]. Most critically, our models treat morphogenesis as a purely viscoelastic deformation, lacking any mechanism that would produce permanent changes in tissue lengths during morphogenesis, e.g. that tissue architecture would remain unchanged by plastic deformation, shear slippage at interfaces, unrecoverable creep, or cell rearrangement. Although Luu et al [53] have argued that the *Xenopus* embryonic epithelium displays a long-term “pseudo-elasticity,” consistent with our models, we suspect that the apparent long-term elasticity they observe may be an artifact of wound-induced contractions [48]. At present however, mechanical measurements presented here and elsewhere [38], [40], [53], [54] do not provide sufficient constraints on plasticity, unrecoverable creep, or the mechanics of cell rearrangement to incorporate these phenomena into our model. Finally, our models invoke several as yet untested assumptions regarding the relationship among the force of induced contractions, endogenous forces driving blastopore closure, and the time dependence of forces driving blastopore closure. Although such complications would change the quantitative predictions of the models, they would not alter the conclusion that the time-dependence of the forces and the time-dependence of deformation could strongly affect the sensitivity of morphogenesis to variation in developmental rates.

Surprisingly, our viscoelastic deformation model predicts that toxins or mutations which alter cell viscoelasticity or the time dependence of force generation will alter the temperature dependence of morphogenetic rates and the temperature sensitivity for defects in opposite ways. For example, blebbistatin treated embryos have more fluid-like tissues (higher β; Fig. 1E). Therefore we would expect that the rate of blastopore closure should increase more slowly with temperature in blebbistatin treated embryos, but the permissive temperature range should be narrower because the reduced temperature sensitivity of tissue movements should lead to greater asynchrony between tissue movements and patterning (Fig. 3B). The cellular processes driving closure are likely to begin uniformly around dorsalized or ventralized embryos, therefore such embryos should exhibit a more step-like onset of the forces driving blastopore closure than normal embryos. Hence, we would also expect reduced temperature dependence of rates of blastopore closure, but a narrower permissive temperature range, in dorsalized/ventralized embryos than in normal embryos. Future studies should investigate whether the model accurately predicts teratological effects of interactions among temperature and other perturbations.

A surprising finding was that the durations of stochastic actin contractions, whose dynamics are considered major contributors to morphogenesis [55], were much less sensitive to temperature than either morphogenetic rates or stimulated, force-generating cellular contractions. Therefore we suspect at least two regulatory mechanisms control the temperature dependence of cytoskeletal dynamics. Actomyosin contractility in the cell cortex observed by confocal microscopy correlated qualitatively with the changes in the speed of current-induced contractions in the micro-aspirator. This relationship was best observed in the higher temperature regime; however, at the low temperature regime there appeared to be little correspondence between the duration of F-actin contractions and induced contractions. Large numbers of short duration contractions in the cortex at 16°C suggest that actomyosin contractility may become decoupled from the long-duration contractions that produce tension in the embryo. Formally, it is possible that we may have under-counted large numbers of short duration contractions at the highest temperature, however, predicted short duration contractions were not observed at intermediate temperatures.

These findings suggest that molecular controls on actomyosin contractility function differently at high and low temperatures. It is unclear how these changes in cytoskeletal dynamics might work to maintain levels of force production and mechanical properties from 16 to 26°C, or whether these dynamics contribute to the failure of morphogenesis outside that range.

By considering how organisms tolerate the forms of environmental variation they have evolved to withstand in nature, we gain new insights into the mechanisms of development. Our models suggest that biomechanical parameters – viscoelasticity and the time dependence of force generation – have a major role in determining the temperature dependence of development. However, it is not the role we first expected. By modulating the synchrony of morphogenesis and patterning, these parameters might influence the evolution of heterochrony and affect the temperature dependence of developmental defects. Our study suggests that embryos tolerate some variation in the relative rates of patterning and mechanical tissue movements, but we hypothesize that increasing levels of asynchrony may lead to gastrulation defects or congenital birth defects. Further work needs to be done to test the predictions of our models, and to test the relationship between short-time scale induced force generation and endogenous forces driving morphogenesis. Additional studies will be needed to extend the experimental work here to temperature ranges that induce developmental defects and develop complimentary models that provide insights into the critical processes that break down under these conditions and increase the risk of birth defects in real populations of vertebrate embryos.

## Methods

### Ethics statement

Animals used in this study were treated according to an IACUC approved protocol issued to Dr. Davidson (#: 0903349; Assurance #: A3187-01) which has been reviewed and approved by the University of Pittsburgh Institutional Animal Care and Use Committee. Embryos were collected and cultured as described previously [38], and kept at 15°C until late blastula stage (stage 9, [33]).

### Micro-aspiration and electrical stimulation

Micro-aspiration was carried out similarly to our previous approach, using a 125 µm diameter channel cast in polydimethylsiloxane [38], however the chambers were miniaturized (to 23×34 mm) for drug and temperature experiments. Temperature control was done using two aluminum tubes mounted within the polycarbonate body of the microaspirator. The temperature of fluid running through the tubes was controlled using a recirculating chiller (ThermoCube, Solid State Cooling Systems; Wappingers Falls, NY). Because we could not have metal-media contact, temperature equilibration took up to 10 minutes and had to be adjusted manually to within ±0.25°C. Temperature was monitored using a thermistor (Quality Thermistor, Inc. QTMB-14C3) mounted in the media, no further than 5 mm from the embryo. Temperatures were recorded using a USB Thermistor measurement system (Robert Owen Inc., Albertson, NY). Pressures were controlled hydrostatically using a programmable syringe pump (New Era Pump, Pump Systems Inc., Farmingdale, NY) that was controlled through a custom VI in LabView 2009 (National Instruments Inc., Austin, TX). Tissue boundaries were tracked automatically within the LabView VI but had to be manually corrected in videos from two embryos.

For the experiment to test whether temperature or blebbistatin affected viscoelastic properties and contractions, one (or in a few cases two) embryos from each clutch was chosen at random for each treatment combination (16 vs 26°C and Blebbistatin vs DMSO carrier control). A total of 6 clutches were used, one per day. Blebbistatin (100 µM, racemic; EMD Millipore, Billerica, MA, USA) and DMSO carrier control media were made fresh each day. Both solutions were made with 1/3-strength Modified Barths Saline [56] to which 8 µl/ml antibiotic-antimycotic (A5955; Sigma-Aldrich, St. Louis, MO) and 2 mg/ml bovine serum albumin (Sigma-Aldrich) was added, with a final concentration of 0.2% w/v DMSO (Molecular Biology Grade; Fisher Biotech,Pittsburgh, PA, USA). The order of Blebbistatin or DMSO treatment was randomized on each day, but in the first runs the 26°C treatments were done prior to the 16°C treatments because of the great difference in developmental rates. If an embryo was damaged, or a video was unusable (due to poor imaging or leakage), a new embryo was selected at random. Embryos were cultured at 15°C until late blastula stage (stage 9), after which they were kept at different temperatures so that embryos could be measured at the same developmental stage. We have not seen morphogenetic defects in embryos transferred from 15 to 26°C or vice versa.

Microaspiration measurements were made at stage 11 on the dorsal quadrant between the blastopore and the equator of the embryo (midway between the animal and vegetal pole). The embryos were held at a low baseline suction (−1 Pa) for 10 min to improve the image of the tissue edge; the suction was then dropped to −11 Pa at −0.82 Pa/s for a 5 minute creep test, after which a 4 ms×2.5 µA (channel positive) electrical pulse was applied to stimulate contraction (Fig. 1; [38]).

Analysis of tissue viscoelasticity and contractions was carried out using custom code in Matlab version R2010a (Mathworks, Natick, MA). A linear viscoelastic model with power-law viscoelasticity [38], [57] was fitted to the aspirated length (L) of the tissue as a function of time (t):
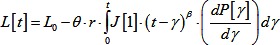
(1)L was measured along the channel centerline. L_0_ is the initial aspirated length; r is channel radius; J[1] is the compliance at 1 second; β is the exponent of the power law creep compliance; and P is pressure in the channel (negative for suction). θ is a proportionality constant that depends on channel wall thickness, and the ratio of tissue thickness to channel radius. For thick tissues and a very thick walled channel, as used here, θ is approximately 0.97 [38], [58]. This model is based on a viscoelastic half-space model [59], incorporating Boltzmann's superposition principle [38], [60], [61]. Previous work showed that the tissue thickness in the aspirated region was always greater than 100 µm (1.6*r; typically much greater) in the measured region of the embryo [62]. Therefore deviations from the half-space model due to finite tissue thickness would be ≤17% [58], [63].

Because the pressure changes occurred as a series of k ramps, this model takes the following form:
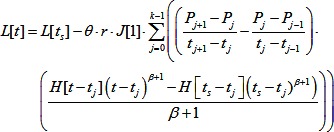
(2)Here, times are relative to the baseline suction, t_S_ is the time at which the creep test began, P_j_ is the pressure at the end of ramp j (P_0_ = P_−1_ = 0), and H is a step function.

Previous work suggested that a model in which apical tension drives electrically induced contraction explains effects of suction pressure on contraction magnitude better than a model in which isotropic contraction stresses occur throughout the aspirated tissue [38]. As described in von Dassow et al 2010, apical tensions are calculated in four steps (Fig. 1B–D). First, the viscoelasticity of the tissue is calculated from aspirated lengths prior to the electrical stimulus. Second, we calculate the “displacements” (m) as the difference between the measured aspirated lengths after stimulation, and extrapolated aspirated lengths. The extrapolation is based on applied pressures and measured viscoelasticity. Third, “equivalent pressure” changes at each time point (Q_k_) are calculated from the displacements and viscoelasticity. These are the changes in suction one would have to apply to mimic the tissue displacements observed during the contraction. To minimize the any effect of the discretization of the contraction forces, a slight refinement to the contraction analysis [38] was implemented. Instead of treating the equivalent pressures as a series of steps, they were treated as a series of ramps. Therefore, the vector of equivalent pressure changes Δ**Q** (each component is the change in equivalent pressure at a given time point) can be calculated from a vector of displacements **m**:
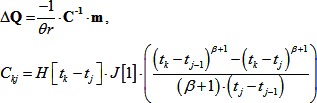
(3)The elements of the matrix C assume ramped changes in stress applied to material with power-law viscoelasticity between t_j_ and t_j+1_. H[x] = 1 for x>0, and H[x] = 0 for x≤0. In addition the displacements were smoothed with a 3-point moving average filter before calculating equivalent pressures to reduce noise in tissue positions that can cause spikes in the equivalent pressures. Finally, the equivalent pressure and estimated radius of curvature (R) of the aspirated tissue were used to calculate the apical tension (f) at each time step, based on the Young-Laplace relation:
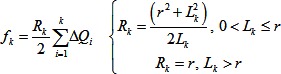
(4)The radius of curvature of the tissue was estimated assuming the tissue approximates a spherical cap.

Fitted viscoelasticity and contraction parameters were analyzed using ANOVAs (Matlab R2010a) with type 3 sums of squares. Temperature and media (Blebbistatin vs DMSO control) were treated as fixed factors, and clutch was treated as a random factor. Two-way interactions were included in the ANOVA model; however the three-way interaction term was not included because there was only one embryo for each treatment-media-clutch combination in most cases.

### Morphogenesis

Time-lapse series of morphogenesis were collected using automated image acquisition software (MicroManager plugin [64] for Image J [65]) to control a motorized stage (Ludl XY and MAC2000 controller, Ludl Electronic Products Ltd., Hawthorne, NY) mounted on a CCD-equipped (Scion Inc, Frederick, MD) inverted microscope (Zeiss Axiovert 100) with a 2.5× lens. Temperatures were maintained using a chamber warmed or chilled by passing fluid through two stainless steel capillary tubes immersed in the media within 1 to 4 mm of the embryos. The temperature was maintained using a recirculating chiller (ThermoCube), and monitored with a thermistor placed in the media as close as possible to the embryos on the bottom coverslip. To minimize temperature variation, a box was placed over the chamber and a thin, closed air space was formed under the chamber using a second coverslip separated from the chamber by a washer. Temperature within the chamber varied by ≤0.4°C with time or position, and usually by ≤0.2°C.

Blastopore closure was filmed in 6 to 8 embryos from each of several clutches at 16° and 26°C. The ratio of the time for blastopore closure or the time for the dorsal-to-ventral progression of involution was measured for each embryo. Because there may be clutch-to-clutch variation in the timing of morphogenesis, the medians of these two parameters were calculated for each clutch. Those embryos which rolled out of view before the completion of blastopore closure were not analyzed. Excluding these embryos did not have a substantial effect on the results. We used t-tests (comparing the set of clutch medians) to test whether clutches incubated at different temperatures differed in the timing of morphogenesis. Data analysis of the timing of morphogenesis was carried out in Microsoft Excel 2010.

Bootstrap analysis [66] of the predictions of the ramp and step models was carried out with custom code (Matlab R2010a). Because the variables in the model come from two experiments, data from the two sets was resampled separately and entered into the equations in Models 2A & B (below). J[1], f, and h always appear together as a product in those equations, so the product of these variables was calculated for each embryo and resampled. Confidence intervals were estimated using the percentile method.

### Actin dynamics

In order to track F-actin dynamics, fertilized *Xenopus laevis* eggs were injected at the 1-cell stage with synthetically transcribed mRNA encoding the actin binding domain of moesin coupled to EGFP (moeGFP) [49], [67]. Embryos were cultured to late blastula or early gastrula stages in 1/3-strength Modified Barths Saline [56]. Animal cap ectoderm was dissected from staged embryos and gently compressed under glass cover-slip fragments so that the basolateral surface of deep cells faced fibronectin-coated glass mounted in a custom chamber designed for stable temperature control. The chamber and connected temperature controlled circulating water bath was identical to the one used for time-lapse imaging of whole embryos. Fluorescence images were optimized [68] and time-lapse sequences were collected using a laser scanning confocal microscope mounted on an inverted compound microscope (Leica TCS SP5; Leica Microsystems, Bannockburn IL). Time-lapse sequences were subsequently analyzed within image processing software (ImageJ) where the starting and ending frame of each contraction were identified. Contraction durations were analyzed using ANCOVA in Matlab R2010a. Temperature was included as a continuous variable, incorporating linear and quadratic terms in the ANCOVA [69]. To account for the possibility of embryo-to-embryo and clutch-to-clutch differences, clutch and explant were included in the ANCOVA as random factors (explant nested within clutch; [69]). Data was plotted using SigmaPlot (SPSS Inc., Chicago, IL).

### Models

**Figure 5:**
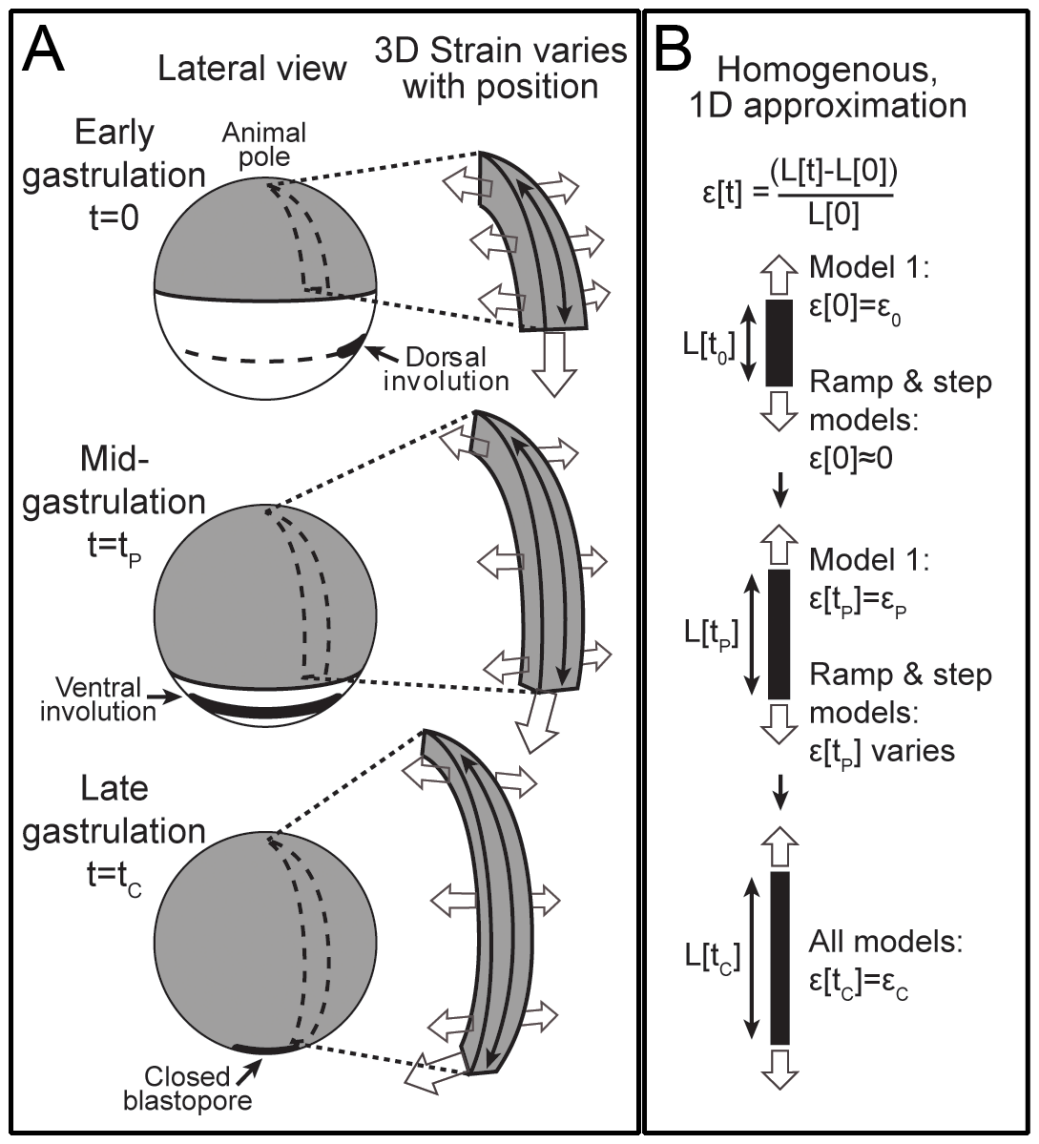
Model schematic. (A) Diagrams of blastopore closure from the lateral side. The ectoderm and neurectoderm (gray) spreads over the embryo during gastrulation. Involution begins on the dorsal side at t = 0, and begins on the ventral side at t_P_; the blastopore closes at t_C_. In the generalized model (Model 1) we assumed all morphogenetic durations (t_P_, t_C_, etc) changed by the same proportion with temperature. In the step and ramp models (Models 2A & B) t_P_ is used, as an estimate of the timing of cell behaviors that exert morphogenetic forces, to predict t_C_. A strip of tissue (A, to right of each whole embryo schematic) experiences spatially and temporally varying stresses (open arrows; stresses from deep tissues not shown), which elongate it and change its shape. We approximate this deformation as uniform stretching of a strip of material (B). The generalized model (Model 1) assumes temperature only affects the speed of morphogenesis, therefore each morphogenetic event occurs at fixed, but unspecified strains (ε_P_,ε_C_,…). In the step and ramp models (Models 2A & B) the main forces driving blastopore closure begin near the start of ventral involution (so ε[0]≈0), and blastopore closure occurs at a fixed strain (ε[t_C_] = ε_C_); however, the strain at t_P_ varies with temperature.

In each of the following models (Models 1 and 2) we approximate the complex, non-linear, three dimensional (3D) deformations of morphogenesis as a one-dimensional (1D), spatially homogenous, linear viscoelastic process to focus on the effect of temperature (Figure 5; and Text S1.1).

### Model 1: Generalized model

We started by assuming that the process of development is identical at different temperatures except that every process is accelerated or decelerated by the same amount for a given change in temperature. This implies that we can define a time scale - developmental time (τ) - that is proportional to clock time (t) via a factor (α) that is a function of temperature (T) so that a developmental event occurring at developmental time τ at temperature T_1_ would occur at the same developmental time at all other temperatures:
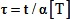
(5)


Strain is a non-dimensional measure of deformation. For extension or compression, strain can be measured as Ln[L/L_0_], where L is the deformed length of the material, and L_0_ is the undeformed length. Since all deformations are identical at a given developmental time τ, all strains (ε^*^) are identical at a given developmental time:
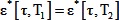
(6)


For a highly simplified linear, small deformation, one dimensional model [60], this implies:
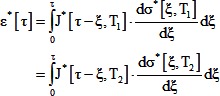
(7)Here, J^*^ and σ^*^ are the creep compliance and stress as functions of developmental time (eqn. 5). Creep compliance is a material property that relates the applied stress (force per unit area) to strain. For an elastic (spring-like) material, compliance is the inverse of the elastic modulus (Young's modulus for tension or compression).

We further assume that the stress at any developmental time T_2_ is proportional to the stress at the same developmental time at T_1_:
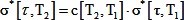
(8)This implies:
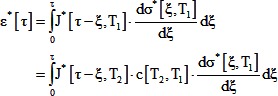
(9)The Laplace transform of equation 9 gives:
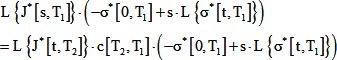
(10)This expression simplifies to:
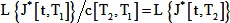
(11)Taking the inverse Laplace transform gives:
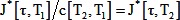
(12)Given our definition of developmental time, the creep compliance as a function of clock time (J[t, T]) is related to J*[ô,T] as follows:
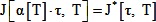
(13)This constrains the form of the temperature dependence of creep compliance, and the temperature dependence of force generation: it exhibits time-temperature superposition [31], with identical time scaling as morphogenesis.

The *Xenopus laevis* gastrula epithelium exhibits power law creep compliance [38]:
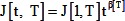
(14)Substituting the developmental time into equation 14, putting it into equation 12 and rearranging gives:
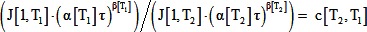
(15)Since J[1,T] and α[T] are independent of time (time is fixed at 1 in J[1,T]), the only way for this expression to be independent of time is if β is independent of temperature. Therefore, this simplified model predicts that β is independent of temperature.

In addition, the coefficient, J[1,T], the stress ratio c, and the temperature dependence of morphogenesis are related by:
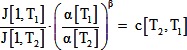
(16)This predicts that if development slows down at lower temperatures, and the coefficient J[1, T] does not change with temperature, then morphogenetic forces should be weaker at lower temperatures. For T_2_ = 16°C, and T_1_ = 26°C, the ratio of α's approximately was approximately 1/3, the ratio of J[1]'s was 1, and β is approximately 0.2. Therefore for contractions, we would expect c, the ratio of the peak apical tension at 16°C to the peak tension at 26°C, to be approximately 0.8, rather than the observed value of approximately 1.4.

### Models 2A and 2B: Specific models

These models retain the simplifying assumption that the complex 3D, large deformation process of morphogenesis can be approximated by a simplified 1D, linear viscoelastic model (Fig. 5; Text S1.1 includes a justifications for this approximation). One outcome of our approximation approach is that we characterize the progress of blastopore closure by a single parameter that scales approximately with the strain field throughout the whole system. This parameter, ε, behaves as the strain in the linear 1 dimensional models below. Because all of the complex deformations during blastopore closure are indexed to this parameter, blastopore closure occurs at a particular value of this “strain,” ε_C_.

### Model 2A: Step model

A step of stress starting at t = 0 would give strain (ε) as follows, where σ is the step stress, and the other variables are as described for model 1 (Generalized Model):
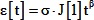
(17)Solving for the time, t_C_, to reach the level of strain, ε_C_, needed to close the blastopore gives:
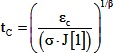
(18)With ε_c_ the same at each temperature, we can substitute 17 back into 18 to determine how t_c_ varies with temperature.
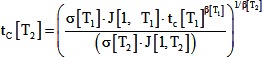
(19)We assume we can express σ in terms of the measured contraction forces. We assume that blastopore closure is driven by pulses of force produced as individual cells intercalate. The average force will be proportional to the force per pulse (f), multiplied by the average number of pulses occurring at a time. The average number of pulses occurring at a time equals the pulse duration (h) multiplied by the rate of pulse initiation. We assume that the pulse duration (h) and force (f) change with temperature in the same way that duration and force of induced contractions change. Because involution involves localized deformations, its dorsal-to-ventral progression should closely follow the initiation times of the cell behaviors that drive it. Therefore, the time (t_p_) it takes for dorsal-to-ventral progression of involution should scale with temperature similarly to the timing of cell behaviors, such as force pulses. Therefore, we assume the rate of force pulses scales inversely with t_p_. These assumptions imply the following, with k a constant of proportionality:
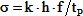
(20)Substituting this into equation 19 and dividing by t_p_ gives an expression predicting how R_CP_ (the ratio t_c_ to t_p_) varies with t_p_, which changes with temperature:
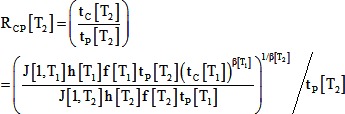
(21)If we assume that f, J[1], and β are independent of temperature (based on the lack of statistically significant effects), and that h is proportional to t_P_ (both biochemically-controlled durations change the same way with temperature), this simplifies to the following:
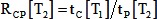
(22)


### Model 2B: Ramp model

**Figure 6:**
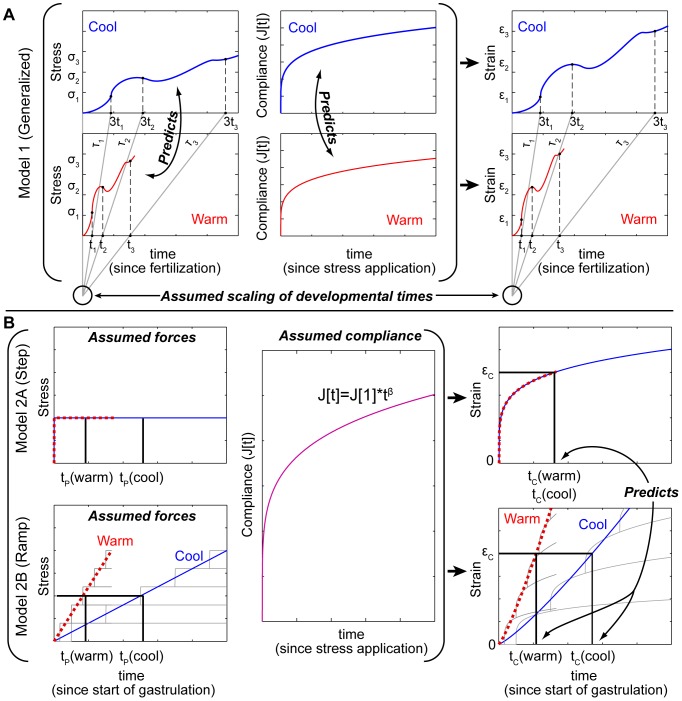
Differences among models. Hypothetical stress (left), creep compliance (middle), and deformation (strain, ε; right) in the tissue. (A) The generalized model (Model 1) assumes the relative timing (ô) and the strains, of all events (1, 2, 3,…) are independent of temperature (cool (blue) vs warm (red)), as in a movie played faster. The generalized model does not specify the developmental course of strain or stress, only that timing scales with temperature. The generalized model predicts how stress and compliance vary together as temperature changes (Model 1, eqn.16). (B) Step and ramp models (Models 2A & 2B). The step and ramp models assume morphogenetic stresses are stepped (top) or ramped (bottom) with time. For a step stress (upper), the change in t_P_ with temperature does not affect the time t_C_ to reach strain ε_C_ (when the blastopore closes) because peak stress and compliance are unchanged (Model 2A, eqns. 21–22). A ramp is the sum of stress increments (gray lines; bottom left). Stress timing (hence the slope of the ramp) scales with t_P_, and therefore with temperature (red, warm; blue, cool). The time t_C_ varies with t_P_ (and therefore temperature) for the ramp model (upper; Model 2B, eqns. 25–26), because strain increments follow the change in timing of stress increments (gray lines).

A ramp of stress starting at t = 0 would give strain (ε) as follows, where w is the slope of the ramp in stress, and the other variables are as described before:
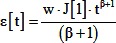
(23)We make the same assumptions regarding the force as in the step model, but with the addition that the frequency of force pulses increases with developmental time with a constant slope. As in the step model, the rate of pulses at any given developmental stage should vary inversely with t_p_. In addition, the time it takes to get to that stage varies directly with t_p_. Therefore, slope of the ramp in stress should be as follows:
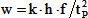
(24)Substituting and rearranging as in the step model gives the following expression for R_CP_, where each parameter is assumed to be a function of temperature:
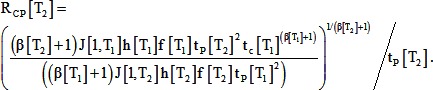
(25)If we assume that f, J[1], and β are independent of temperature, and that h is proportional to t_P_, this simplifies to the following:
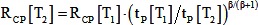
(26)In summary, differences among the models are illustrated graphically in Figure 6. The generalized model (Model 1) makes *no* assumptions about the time course of morphogenetic forces; the step and ramp models *assume* specific time courses of morphogenetic forces (stepped or ramped; Models 2A & B). The generalized model *assumes* that the relative durations of morphogenetic events (e.g. the ratio, R_CP_, of t_C_ to t_P_) do not vary with temperature; the step and ramp models *predicts* the changes in relative durations (R_CP_) with temperature. The generalized model *predicts* the temperature dependence of compliance and stress magnitude; the step and ramp models take these as inputs.

## Supporting Information

Movie S1 **Gastrulation at 16° and 26°C.** This movie shows gastrulation at 16°C (left) and 26°C (right) starting at the late blastula stage, ending after blastopore closure, as the neural plate converges on the dorsal side (top). Embryos are in a vegetal pole view. Movies were rotated so that the dorsal side is towards is at the top of the image, cropped to fit, and contrast was adjusted to optimize the image. Note that the embryo at 16°C was filmed at 2 minutes per frame, while the embryo at 26°C was filmed at 1 minute per frame. For this combined movie their playback rates were adjusted to match, and they were synchronized to the beginning of involution on the dorsal side. Early on in each embryo, the apices of bottle cells contract strongly. This begins on the dorsal side of the embryo, eventually forming a dark ring of cells with narrowed apices around the blastopore. Soon after the ring fully forms, the superficial layer of tissue outside the blastopore on the dorsal side begins to involute (“dorsal involution”), appearing to roll inwards over the blastopore lip and inside the embryo. Involution progresses around the blastopore, until it occurs on the ventral side as well (“ventral involution”). This time difference is t_p_. The blastopore finally closes (“blastopore closure”) sometime after involution begins on the ventral side, but involution continues after blastopore closure. Note that the ectoderm and mesoderm move vegetally while the ring of bottle cells contracts, before superficial involution begins. However, we defined the time for blastopore closure (t_c_) based on the beginning of involution because it is a much more clearly marked time point.
(MOV)

Movie S2 **F-actin dynamics at low (16°C) and high (26°C) permissive temperatures.** Confocal time lapse sequences of moe-GFP within the basal cell cortex of deep ectodermal cells from gastrula stage embryos. Both sequences were collected with the same confocal settings and magnification at 10 second intervals. Sequences appear to fade at times due to thermal drift which was corrected manually during the collection. The scale-bar indicates 10 µm.
(MOV)

Text S1 **Supporting text.** The supplemental text includes a justification for using a linear, one dimensional approximation in models of blastopore closure (S1.1) and a description of an unusual example of tolerance to variation in gastrulation (S1.2).
(PDF)
