# Supplementary material for: Biomechanics and the Thermotolerance of Development
Source: PLoS One. 2014 Apr 28;9(4):e95670. doi: 10.1371/journal.pone.0095670 (PMC4002435; doi:10.1371/journal.pone.0095670)
Supplement: Text S1 — Supporting text. The supplemental text includes a justification for using a linear, one dimensional approximation in models of blastopore closure (S1.1) and a description of an unusual example of tolerance to variation in gastrulation (S1.2). (PDF) [file pone.0095670.s003.pdf]

**Supplemental Text**

**pp2-3 Supplemental Text S1.1: 1D, small deformation approximation.**

**p4 Supplemental Text S1.2: An example of tolerance to variation in gastrulation.**

**p8 Supplemental references.**

**Supplemental Text 1.1: 1D, small deformation approximation.**

Blastopore closure is a complex, large-deformation problem (hence geometrically non-linear), involving materials that are likely to display non-linear material properties at sufficiently large deformations [1,2]. Furthermore material properties and force generation vary spatially [2]. A full model would require a very large number of parameters, many of which are unknown, and most of which are poorly characterized. Those parameters that have been measured are highly variable [1,2,3,4]. We seek a simplified model, with as few parameters as possible, to explore the effects of variation in viscoelasticity and timing of force generation on the temperature dependence of morphogenesis.

Here we argue that a simple linear, scalar (1D) model is a reasonable first approximation given the number of unknown parameters, and high levels of mechanical variation. This model captures expected behaviors such as: 1) increased forces lead to increased deformation; 2) usually (though not always) the rate of deformation declines with time after application of a load; and 3) compliance measured in the microaspirator should correlate with tissue responses to stresses driving blastopore closure (i.e. tissues that appear softer in microaspiration should behave as though they are softer during blastopore closure). We also expect that if the tissue behaves like a solid in the microaspirator, it will behave like a solid during blastopore closure. Similarly if it behaves like a fluid in the microaspirator, it should also behave like a fluid during blastopore closure.

Consider the full 3D, non-linear, large-deformation model. The strain tensor ($\mu_{ij}$) at time t and position X={x, y, z} is a function of the stress tensor σ and compliance tensor C:

(eqn. S1) $$\mu_{ij}[X,t] = f\left[C_{ijkl}[X,\gamma], \sigma_{kl}[X,\gamma], t\right]$$

We assume zero strain at the beginning of gastrulation. C and σ are functions of both time and position in the embryo, and f is a function of C and σ over all points in time prior to t (represented by γ). Let us take $\sigma_{kl}=s_{kl}*\Theta$, where Θ is a scalar, with f going to zero as Θ goes to zero (i.e. when stress is zero at all times). Then approximating with a Taylor series around Θ=0,

(eqn. S2) $$\mu_{ij}[X,t] = \frac{df}{d\theta}\theta + \omega[\theta]$$

Here ω[θ] are higher order terms in the series. The term (df/dθ)θ is the linear, small deformation viscoelastic model. So we can write:

(eqn. S3) $$\mu_{ij}[X,t] = \theta\left(\int_0^t C_{ijkl}[X,t-\gamma]\frac{ds_{kl}[X,\gamma]}{d\gamma}d\gamma\right) + \omega[\theta]$$

Furthermore, we can split $s_{kl}$ into two terms:

(eqn. S4) $$s_{kl}[X,t] = z_{kl}[X]v[t] + y_{kl}[X,t]$$

Here $z_{kl}$[X] is the time averaged value of $s_{kl}$ at position X, v[t] is a scalar function of time, and $y_{kl}$[X, t] are the deviations from $z_{kl}$[X]v[t]. We can choose v[t] in a way that minimizes the contribution of y[X, t]$_{kl}$ to the strain (it is not important how to do this, only that there exists a choice of y and v that would maximize the contribution of z). Hence,

(eqn. S5)

$$\mu_{ij}[X,t] = \theta\left(z_{kl}[X]\int_0^t C_{ijkl}[X,t-\gamma]\frac{dv[\gamma]}{d\gamma}d\gamma + \int_0^t C_{ijkl}[X,t-\gamma]\frac{dy_{kl}[X,\gamma]}{d\gamma}d\gamma\right) + \omega[\theta]$$

Both ω and y are unknown even to sign, and y is chosen so that its contribution is as small as possible. Therefore, it is reasonable to take as a first approximation, the linear model:

(eqn. S5) $$\mu_{ij}[X,t] \approx \theta z_{kl}[X]\int_0^t C_{ijkl}[X,t-\gamma]\frac{dv[\gamma]}{d\gamma}d\gamma$$

The compliance and strain are still 2nd and 4th order tensors. If we assume that $C_{ijkl}$ can be approximated as the product of a scalar valued term (J) depending on time, and a tensor ($c_{ijkl}$), we get:

(eqn. S6) $$\mu_{ij}[X,t] \approx c_{ijkl}[X]\theta z_{kl}[X]\int_0^t J[\,t-\gamma]\frac{dv[\gamma]}{d\gamma}d\gamma$$

(Alternatively one could use a similar division into the sum of two tensors as used for $s_{ij}$.) This means that $\mu_{ij}$[X, t] must also be approximately separable into products of a spatially varying term ($g_{ij}$[X]) and a temporally varying term (ε[t]) in the same way. All the spatial variation can be separated into one set of terms, and we can simplify to a scalar equation equivalent to our linear model. The unknown proportionality term (K) cancels out of the final model:

(eqn. S5) $$\varepsilon[t] \approx K\int_0^t J[\,t-\gamma]\frac{dv[X,\gamma]}{d\gamma}d\gamma$$

The scalar ε is now the only parameter that characterizes the progression of gastrulation, so we can map morphogenetic events (deformation states) to ε[t]. Hence, blastopore closure begins and ends at a particular values of ε (0 and $\varepsilon_c$ respectively).

Clearly, we have cut out a lot of important processes and parameters to get to this point. Furthermore, we have not accounted for changes in compliance over development [2,3]. However, given the unknowns, and the variation in the known parameters, this linear, scalar-valued model is a reasonable first approximation. It is also minimal: all parameters other than those that whose importance we wish to determine (v[t]), can be tied to measurements (J[t]), or cancel out (K).

## Supplemental Text 1.2: An example of tolerance to variation in gastrulation.

A remarkable illustration of embryos' capacity to tolerate morphogenetic variation occurred in one clutch (a batch of embryos collected from the same female at the same time). In this clutch (Figure S1) gastrulation took a very different path than in others (7 of 8 embryos). Bottle cell formation began normally. However, at the point when superficial involution would normally begin at the dorsal blastopore edge, a tongue of material (most likely presumptive notochord) extended out across the blastopore from the dorsal side. Superficial involution then began at a point further up the dorsal side, and blastopore closure occurred over the tongue of material. Despite its abnormal trajectory, blastopore closure completed successfully and the resulting embryos looked essentially normal at later stages (neurula and early tadpole). Because blastopore closure was so unusual in this clutch, these results were not analyzed for shifts in developmental timing, yet they provide a clear example of how robust morphogenesis can be.

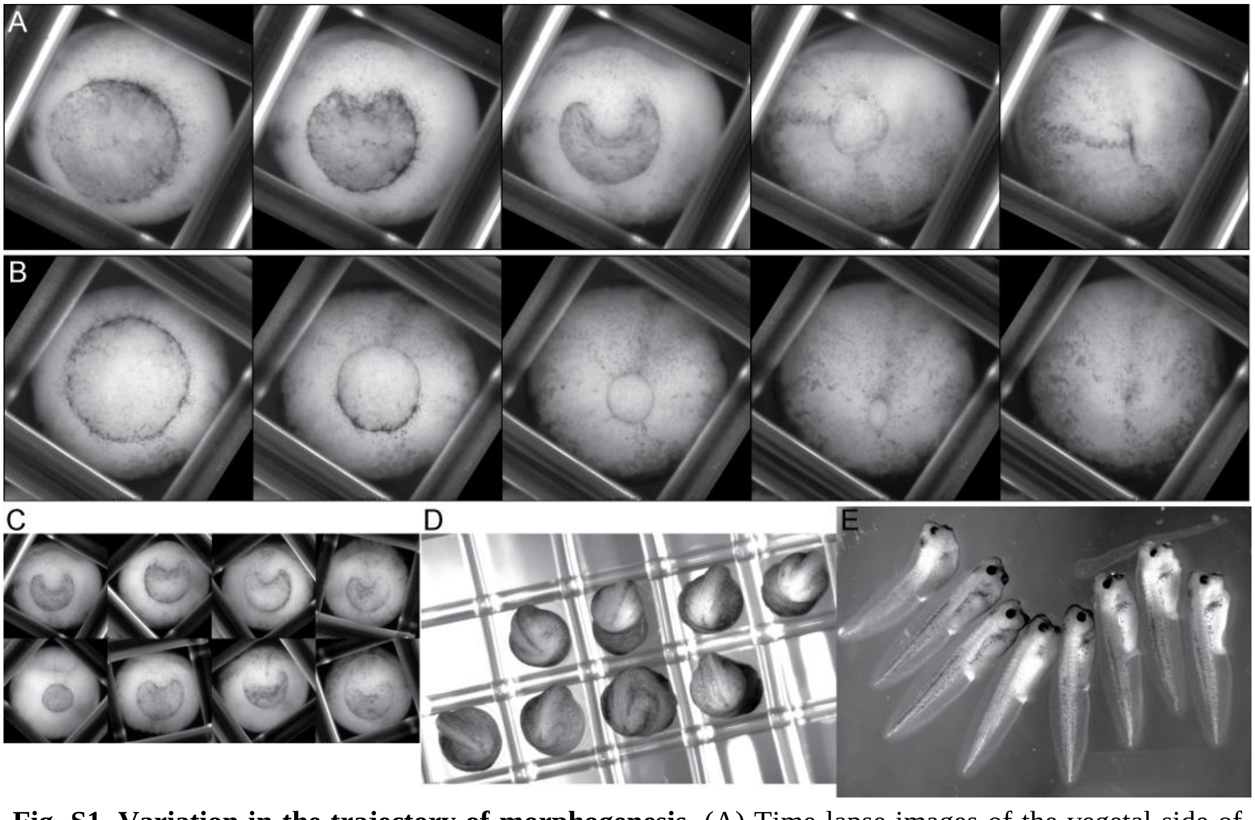


**Fig. S1. Variation in the trajectory of morphogenesis.** (A) Time lapse images of the vegetal side of an embryo in a clutch with an unusual trajectory of morphogenesis. A tongue of material extended over the blastopore from the dorsal side at the time when normal embryos (B) would be undergoing dorsal superficial involution. Images in A and B are at hourly intervals from bottle cell contraction (left) to blastopore closure (right) at 26°C. (C) The same time point midway through blastopore closure in all 8 embryos from this unusual clutch. (D) Posterior ends of the same eight embryos at neurula stages had completed blastopore closure. (D) The same eight embryos at tadpole stages showed only minor defects. Contrast and brightness were adjusted to optimize images; images in A - C were rotated dorsal side up. B shows embryo from Figure 2A.

**Supplement References:**

1. von Dassow M, Strother JA, Davidson LA (2010) Surprisingly simple mechanical behavior of a complex embryonic tissue. PLoS One 5: e15359.

2. Zhou J, Kim HY, Davidson LA (2009) Actomyosin stiffens the vertebrate embryo during critical stages of elongation and neural tube closure. Development 136: 677-688.

3. von Dassow M, Davidson LA (2009) Natural variation in embryo mechanics: gastrulation in *Xenopus laevis* is highly robust to variation in tissue stiffness. Dev Dyn 238: 2-18.

4. Kalantarian A, Ninomiya H, Saad SM, David R, Winklbauer R, et al. (2009) Axisymmetric drop shape analysis for estimating the surface tension of cell aggregates by centrifugation. Biophys J 96: 1606-1616.
